# Antenatal subpial hemorrhage diagnosed by fetal magnetic resonance imaging: imaging spectrum and autopsy findings

**DOI:** 10.1007/s00247-025-06353-9

**Published:** 2025-07-30

**Authors:** Stephanie Libzon, Debora Kidron, Uri Erlik, Karina Krajden-Haratz, Moran Hausman-Kedem, Liat Ben Sira

**Affiliations:** 1 Pediatric Neurology Institute, Dana-Dwek Children’s Hospital, Tel Aviv Medical Center, 6 Weizmann Street, Tel Aviv, 6423906 Israel; 2 Department of Physical Therapy, School of Health Professions, Gray Faculty of Medical and Health Science, Tel Aviv University, Tel Aviv, Israel; 3 Department of Pathology, Meir Medical Center, Kfar-Saba, Israel; 4 Gray Faculty of Medical and Health Science, Tel Aviv University, Tel Aviv, Israel; 5 Division of Ultrasound in Obstetrics and Gynecology, Lis Maternity and Women’s Hospital, Tel Aviv Medical Center, Tel Aviv, Israel; 6 Division of Ultrasound in Obstetrics and Gynecology, Mayanei Hayeshua Medical Center, Bnei Brak, Israel; 7 Department of Radiology, Division of Pediatric Radiology, Dana-Dwek Children’s Hospital, Tel Aviv Medical Center, 6 Weizmann Street, Tel Aviv, 6423906 Israel

**Keywords:** Autopsy, Fetal, Magnetic resonance imaging, Prenatal diagnosis, Subpial hemorrhage

## Abstract

Subpial hemorrhage is a rare intracranial hemorrhage typically described in neonates. We report the first prenatal diagnosis of subpial hemorrhage in a 28-year-old primigravida, defined on fetal magnetic resonance imaging **(**MRI) by its hallmark cortical inward depression (“cortical buckling”) and restricted diffusion on the apparent diffusion coefficient map, and later confirmed by autopsy. This case implicates intrinsic fetal factors—rather than birth trauma or neonatal asphyxia—in subpial hemorrhage pathogenesis and highlights the critical role of fetal MRI in distinguishing subpial hemorrhage from other fetal hemorrhages, with important implications for prenatal counseling and perinatal management.

## Introduction

Subpial hemorrhage is a rare form of intracranial hemorrhage usually occurring in term neonates [1] and its exact pathophysiology is still subject to debate. It may be caused by rupture of cortical vessels that are not encased by a leptomeningeal sheath due to injury to the glial limitans, which is the outermost layer of astrocyte foot processes in the neocortex, resulting in bleeding into the potential space between the pia mater and glial limitans. This causes a mass effect and compression of the subpial veins, with local venous congestion and hypertension, resulting in focal cortical or subcortical venous infarction, and adjacent cortical damage [2].

Some studies have suggested that birth trauma may play a role in subpial hemorrhage [1, 3]. In addition, most neonates with subpial hemorrhage were born by vaginal delivery, suggesting a possible link to the mechanics of labor. However, most births are uneventful, and the evidence is not conclusive. Other risk factors have been proposed, including fetal distress and neonatal asphyxia, cardiorespiratory failure, fetal head molding, coagulation disorders, venous sinus compression, variations in intracranial pressure, and incomplete regression of the primary vascular network [2, 4].

Although first described in 1972 by autopsy, subpial hemorrhage was precisely defined with the use of various sequences in magnetic resonance imaging (MRI) only in 2004 [5], unraveling the spectrum of parenchymal involvement with distinct MRI features. A focal mass effect, due to a “compartment syndrome,” secondary to an absent ability to resorb the bleed, causes a unique imaging feature of cortical inward depression (cortical buckling) and patchy cortical and subcortical injury [2]. The consistent presence of an egg-shaped subpial bleed with underlying restrictive, focal, cerebral cortex, created a distinct MR imaging pattern, resembling the yin-yang symbol in Chinese philosophy [2].

All subpial hemorrhages, to date, have been described solely postnatally, usually in neonates [3, 6, 7], but they have also been described in adults. We report an antenatal diagnosis of subpial hemorrhage shedding the light on the presumed pathophysiology.

Approval to conduct this study was obtained from the institutional review board (TLV-131–24). Written informed consent was obtained from the patient. No funding was received for conducting this study.

## Case presentation

A 28-year-old woman in her first pregnancy was referred to our fetal MRI unit after a third-trimester ultrasound revealed fetal brain hemorrhage.

Maternal history was positive for celiac disease under a gluten-free diet. Routine pregnancy follow‐up, including nuchal translucency, first‐trimester triple test, and first‐ and second‐trimester anatomy screening scans, was considered normal, with no evidence of hemorrhage or vascular malformation on Doppler sonography (last follow-up at 27 weeks gestational age). Amniocentesis was performed with a normal chromosomal microarray for a male fetus.

**Fig. 1:**
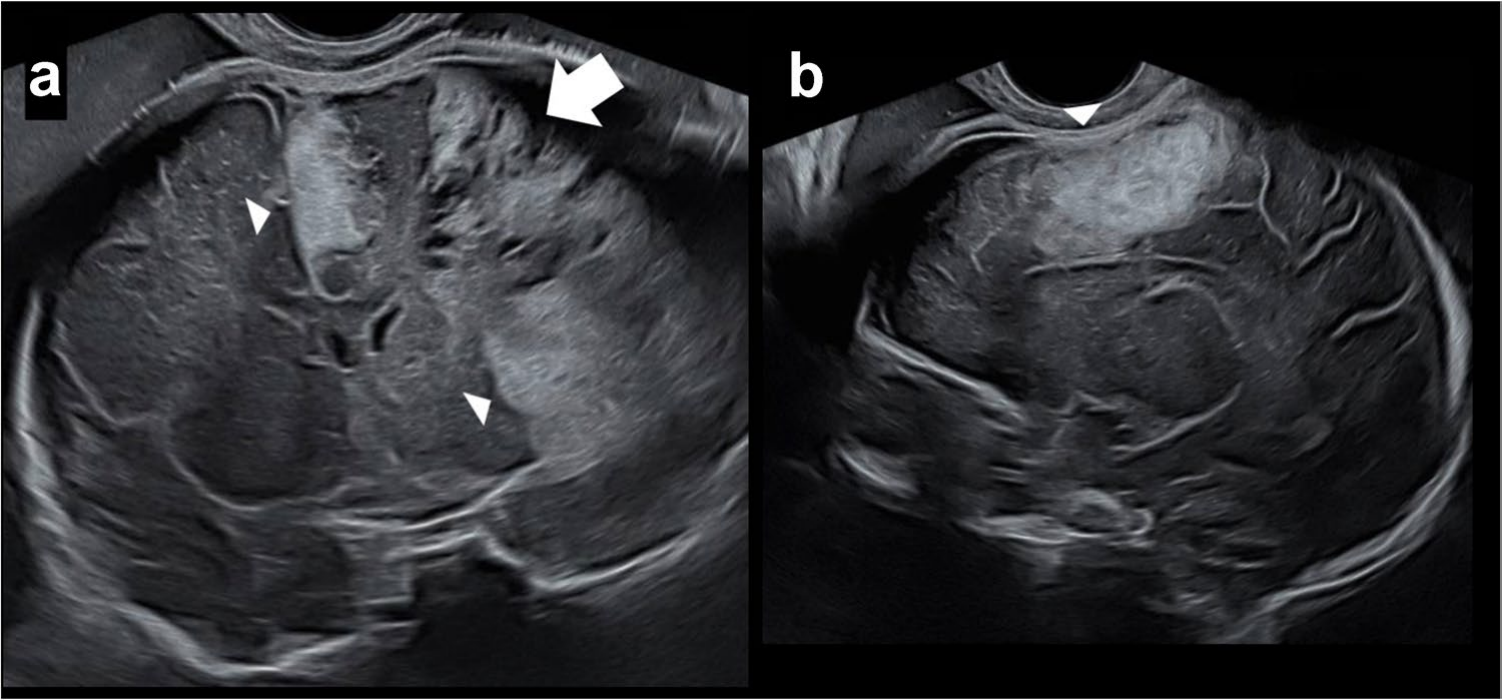
Ultrasound images of the brain in a male fetus at 32 + 4 weeks gestational age demonstrate hemorrhage. **a** Coronal image shows diffuse parenchyma and extra axial echogenicities, fronto-parietal lobes (*arrowhead*), with cystic evolution (*arrow*) consistent with hemorrhages at different stages. **b** A para-sagittal image shows echogenic parenchyma hemorrhage (*arrowhead*)

Routine ultrasound (US) at 32 + 4 weeks gestational age revealed a large hyperechogenic area in the left frontal lobe, related to massive hemorrhage, without midline shift. Hemorrhage was also noted above the ipsilateral cerebellum (Fig. 1). A differential diagnosis of parenchymal or extra axial hemorrhage was raised.

The patient was sent for a fetal MRI, performed the next day, at 32 + 5 weeks gestational age. Imaging revealed a large extra axial collection over the left cerebral hemisphere; a hypointense signal in T2 confirmed as hemorrhage by susceptibility-weighted imaging, with a mild hyperintensity signal in T1. The hemorrhagic fluid collection measured up to 2 cm in maximum depth.

Beneath the hematoma, the underlying brain parenchyma, in the left fronto-parietal-temporal cortex, showed T2 hyperintensity accompanied by diffusion restriction that extended into the left thalamus. Within this parenchymal component area, there were linear, tiny T2 hypointense structures, which suggested the presence of engorged medullary veins. Marked susceptibility to blooming artifacts from hemosiderin deposition was evident in these areas.

**Fig. 2:**
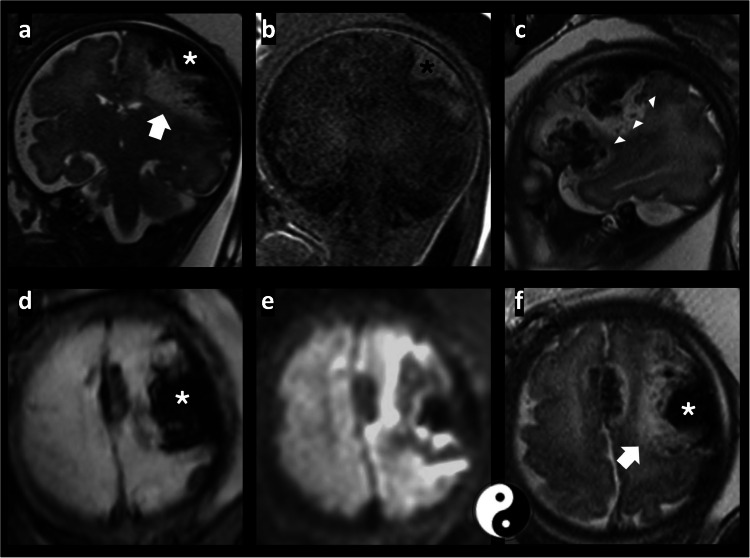
Magnetic resonance images of the brain in a male fetus at 32 + 5 weeks gestational age demonstrate massive subpial hemorrhage. **a** Coronal T2-weighted image shows extra axial hypointense signal, consistent with a front-parietal lobes hemorrhage (*white asterisk*). The underlying cortex demonstrates diffuse hyperintense T2 signal (*arrow*). **b** Coronal T1-weighted image shows hyperintense signal confirming subacute front-parietal lobes hemorrhage (*black asterisk*). **c** Sagittal T2-weighted image shows linear T2 hypointensities within the underlying cortex, suggesting engorged medullary veins (*arrowhead*). **d** Axial susceptibility-weighted imaging shows increased susceptibility-blooming-related artefact from hemosiderin in the cortex and the extra axial areas, rendering them indistinguishable on susceptibility images (*white asterisk*). **e** Axial diffusion-weighted image shows restricted diffusion in the adjacent parenchyma, differentiating the underlying restrictive cortex from the overlying subpial hematoma. **f** Axial T2-weighted image shows hypointense subpial hemorrhage (*white asterisk*), underlying hyperintense cortex (*arrow*) with a typical “yin-yang” appearance: cortical buckling and patchy cortical and subcortical injury

Similar signal abnormalities were noted in the frontal interhemispheric region. A focal mass effect with compression of the adjacent parenchyma and a rightward midline shift was also observed (Fig. 2).

The characteristic combination of a rounded, egg-shaped subpial hemorrhage with underlying restricted diffusion in the adjacent cortex formed a distinct imaging pattern, resembling the “yin-yang” symbol in Chinese philosophy. On apparent diffusion coefficient maps, true restricted diffusion sharply delineated the cortical ribbon and subcortical white matter adjacent to the subpial hemorrhage, consistent with subpial hemorrhage. Additional infra- and supratentorial hemorrhages were also identified. Maternal and fetal testing, including whole-exome sequencing, as well as tests for coagulopathy and antiphospholipid antibodies, yielded negative results.

Based on these findings, the parents were consulted regarding the risk of abnormal neurologic development associated with the multicompartment hemorrhages. They were offered the option of pregnancy termination, which is permitted under national law in late pregnancy for cases involving a high risk of severe neurodevelopmental impairment.

**Fig. 3:**
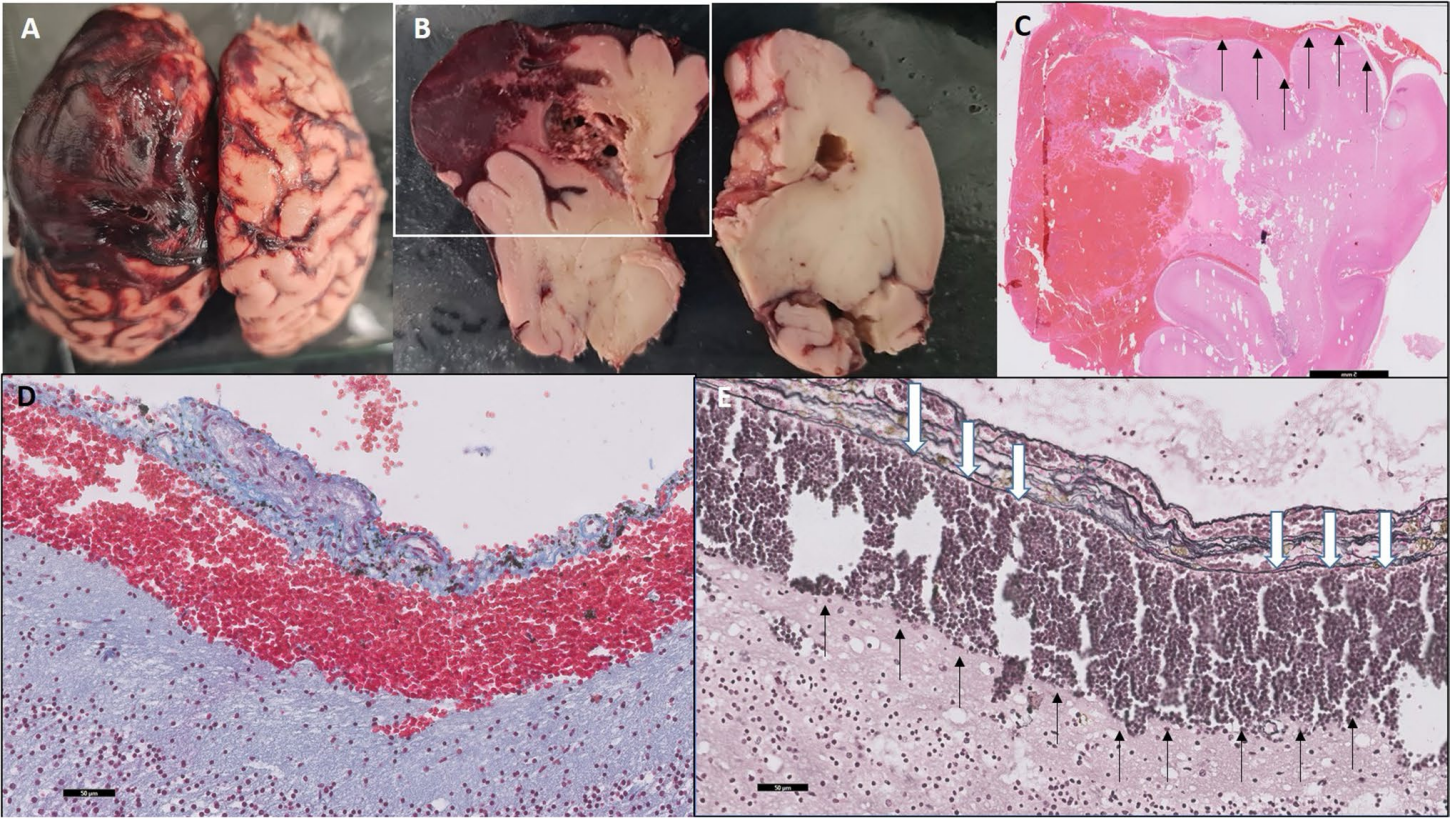
Autopsy at 33 + 1 weeks gestational age confirming the subpial hemorrhage seen in fetal magnetic resonance images. **a** Cerebral hemispheres, superior view. Extensive hemorrhage in the leptomeninges overlying the left hemisphere. **b** Cerebral hemispheres, coronal section. In frame: Hematoma involving cortex and subcortical zones of the left cerebral hemisphere, associated with dissecting hemorrhages throughout white matter. **c** Histologic section by hematoxylin and eosin (H&E) staining, taken from the left cerebral hemisphere, in the coronal plane, parallel to the white frame in **b** (scale bars: 5 mm). In addition to cerebral hematoma and hemorrhages, hemorrhage is seen in the meninges in the superior aspect of the left cerebral hemisphere (*arrows*) (**c**). **d** Masson trichrome stain showing subpial hemorrhage, between the cortex and leptomeninges (scale bars: 50 μm). **e** Reticulum stain highlighting a subpial hemorrhage separating the cortex and glia limitans (*black arrows*) from leptomeninges, which contain reticulum fibers (*white arrows*) (scale bars: 50 μm)

The pregnancy was terminated at 33 + 1 weeks gestational age due to parental request. Autopsy confirmed the presence of a subpial hemorrhage, as observed on MRI, with no evidence of malformed blood vessels or vascular malformation (Fig. 3).

## Discussion

Subpial hemorrhage is a distinct and rare form of intracranial hemorrhage characterized by bleeding between the pia mater and the glia limitans that, until now, has been described exclusively in the postnatal period. To the best of our knowledge, this is the first reported case of subpial hemorrhage diagnosed prenatally and confirmed on autopsy.

The US is limited in visualizing the periphery of the brain, where most subpial hemorrhages occur [2]. Therefore, MRI is required for a more precise evaluation of the full spectrum of subpial hemorrhage, exhibiting distinct imaging features that may aid radiologists and clinicians in its recognition, including cortical inward depression (cortical buckling) and patchy cortical and subcortical injury.

The significance of this case lies in demonstrating how advanced fetal MRI, with its sensitivity to blood products, can reveal the unique pattern characteristic of subpial hemorrhage. This enables differentiation from other, more common causes of fetal hemorrhage.

The plausible pathophysiology of subpial hemorrhage is traditionally associated with postnatal events. Emerging evidence suggests that prenatal factors may play a significant role, especially in cases without perinatal trauma or coagulation disorders [1]. These include placental insufficiency, intrauterine hypoxia, maternal infections, and vascular developmental anomalies, such as incomplete regression of the primary vascular network [2], which may result in structurally fragile vessels that are more prone to rupture under stress [1]. This could explain cases of subpial hemorrhage occurring without identifiable perinatal risk factors, indicating an underlying vulnerability in the cerebral vascular architecture [2]. Venous congestion appears to be a central mechanism in the development of subpial hemorrhage. Medullary vein engorgement or thrombosis has been frequently observed adjacent to subpial hemorrhage, suggesting impaired venous outflow as a critical factor [8].

In our case, there was no indication of maternal trauma, nor was there any evidence of coagulation abnormalities or genetic predisposition that could suggest the previously described mechanisms of injury. Therefore, we can hypothesize that there may be additional, yet unidentified, factors contributing to the increased vulnerability of the vessels in the focal subpial space.

These findings highlight the significance of assessing antenatal conditions when evaluating subpial hemorrhage, especially in neonates without clear perinatal or postnatal risk factors. Additional research is necessary to clarify the specific mechanisms involved and to enhance prenatal detection of fetuses at-risk.

## Data Availability

No datasets were generated or analysed during the current study.
